# Chitin-based Materials in Tissue Engineering: Applications in Soft Tissue and Epithelial Organ

**DOI:** 10.3390/ijms12031936

**Published:** 2011-03-17

**Authors:** Tsung-Lin Yang

**Affiliations:** 1 Department of Otolaryngology, National Taiwan University Hospital and College of Medicine, Taipei, 100, Taiwan; E-Mail: yangtl@ntu.edu.tw; Tel.: +886-2-23123456 ext. 63526; Fax: +886-2-23940049; 2 Department of Otolaryngology, College of Medicine, National Taiwan University, Taipei, 100, Taiwan; 3 Research Center for Developmental Biology and Regenerative Medicine, National Taiwan University, Taipei, 100, Taiwan

**Keywords:** chitin, chitosan, epithelium, soft tissue, tissue engineering, regeneration

## Abstract

Chitin-based materials and their derivatives are receiving increased attention in tissue engineering because of their unique and appealing biological properties. In this review, we summarize the biomedical potential of chitin-based materials, specifically focusing on chitosan, in tissue engineering approaches for epithelial and soft tissues. Both types of tissues play an important role in supporting anatomical structures and physiological functions. Because of the attractive features of chitin-based materials, many characteristics beneficial to tissue regeneration including the preservation of cellular phenotype, binding and enhancement of bioactive factors, control of gene expression, and synthesis and deposition of tissue-specific extracellular matrix are well-regulated by chitin-based scaffolds. These scaffolds can be used in repairing body surface linings, reconstructing tissue structures, regenerating connective tissue, and supporting nerve and vascular growth and connection. The novel use of these scaffolds in promoting the regeneration of various tissues originating from the epithelium and soft tissue demonstrates that these chitin-based materials have versatile properties and functionality and serve as promising substrates for a great number of future applications.

## 1. Introduction

**Figure 1.**
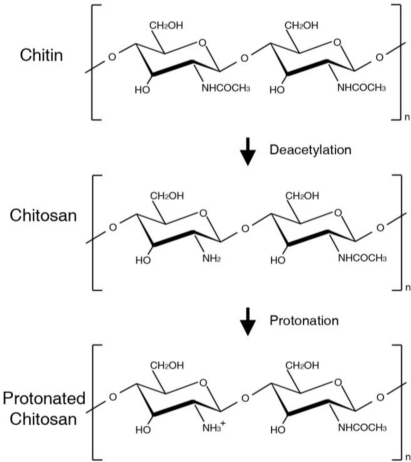
Molecular structures of chitin, chitosan, and protonated chitosan polymer.

Chitin is the second most abundant natural polymer and is commonly found in the exoskeletons of crustacean and insects as well as the cell walls of fungi. Most chitin applications are based on its deacetylated form, chitosan. Chitosan is composed of glucosamine and N-acetyl glucosamine, which are linked in a β (1–4) manner (Figure 1). The molecular weight and degree of deacetylation, which are critical in determining the characteristics of chitosan, depend on the source and production process used [1].

Chitin-based materials have been widely used in the biomedical field. These materials are biocompatible and have been approved for human use [2]. Chitosan can be biodegraded by human enzymes, such as lysozyme that breaks the linkage between acetylated units and degrades chitosan to oligosaccharides [3]. The degradability of chitin-based material is important for scaffold fabrication, because degradation may influence cell behavior and tissue formation of the engineered construct [4]. Chitin-based materials also possess a bactericidal effect. The positive charge of the amino group in the chitosan polymer can react with anions located on the cell wall of bacteria. This reaction causes the bacterial cell to break open and the contents of the bacterial cell leak out. Furthermore, chitosan can bind nucleic acids and interfere with protein synthesis [4,5]. Because of these unique features, chitin-based materials have broad medical utility.

An intriguing and important feature of chitin-based material is its cationic nature. In many physiological situations, chitosan can become protonated and positively-charged (Figure 1). The positive charge of chitosan originates from the protonated amino groups. The protonation usually occurs in acidic environments *in vitro* and *in vivo*, and increases the solubility of chitosan [1,4]. Protonated chitosan can form a complex with many types of negatively-charged molecules, such as growth factors, nucleic acids, and cytokines [6,7]. This feature allows chitosan to recruit and bind bioactive factors from surrounding environments, thereby protecting these factors from degradation and increasing local concentration and efficacy [8–12]. This unique property of chitin-based materials is significant in the modulation of cell behavior during tissue regeneration. In addition, it is possible that positively-charged chitosan interacts with anionic glycosaminoglycans (GAG) and proteoglycans, which are essential components in the extracellular matrix (ECM) found throughout the body. Because a variety of bioactive factors are regulated and are bound to these ECM elements, chitin-based materials capable of interacting with GAG or proteoglycans may be competent in modulating these substantial factors for tissue formation [13].

In the traditional design of biomaterials, bio-inert substrates are favored because of the reduced induction of immune responses; these materials are regarded as biocompatible. With advances in the understanding of cell-biomaterial interactions, the current design of biomaterial scaffolds has focused more on bioactive material systems. Bioactive systems can be established through the development of delivery systems for bioactive molecules, or the fabrication of bioactive scaffolds by using physical or chemical modifications. To this end, chitin-based material is a candidate of choice. Chitosan has been widely applied in drug delivery systems for growth factors, peptides, and bioactive compounds. The simple control over the loading and releasing of bioactive agents renders chitosan an appropriate vehicle for drug delivery [1,14]. When fabricated into tissue-engineered scaffolds, chitin-based materials provide many functional chemical units that can be readily modified for desired bioactivity [15]. In recent years, chitin-based materials have drawn increasing attention because of their potential role in biotechnology and medical applications [1,4].

Tissue engineering employs aspects of molecular biology, cell biology, material technology, engineering, and surgical intervention to develop tissue substitutes to restore the function and architecture of damaged or lost tissues and organs [16–21]. Tissue engineering aims to create new tissues and organs by introducing cells, biocompatible materials, and supportive factors. Cell-seeded biomaterial matrices can be implanted into the body to initiate functional tissue regeneration. To this end, numerous biomaterials ranging from natural to artificial polymers have been investigated to construct scaffolds for tissue engineering purposes. Using chitin-based materials, it is possible to fabricate different types of scaffolds, such as hydrogels, microcapsules, films, porous structures, and fibers. Furthermore, these scaffolds can be modified to yield polymer blends, which are formed with desired structures, chemical features, and mechanical properties. In addition, chitosan is an ecologically-favorable biomaterial with low costs of retrieval and production that can be easily scaled up for industrial use [22]. Together, these excellent features render chitin-based materials optimal for tissue engineering [23,24].

Chitin-based biomaterials have favorable biological properties for tissue regeneration [25–27]. These materials are well known for their potential when used in tissue regeneration of the skeletal system [28–31]. Chitosan possesses properties useful for orthopedic tissue including plastic properties, antibacterial effects, minimal foreign body reactions, increased cell infiltration, and efficacious osteoconduction [29]. Therefore, through precisely controlled production, chitin-based biomaterials can harbor a predictable structure and ideal degradation rates that are suitable for bone graft alternatives. Chitin-based materials can also be incorporated with bioactive factors that are beneficial to osteogenesis [29]. In light of these characteristics, chitin-based materials have been widely used as scaffold substrates for orthopedic tissue engineering in clinical applications [29].

Tissues derived from epithelium and soft tissues are generally responsible for covering, supporting, and connecting organs within the body. These tissues play major roles in maintaining body contours and providing mechanical cushion and protection [32]. Generally, these tissues are constructed with ECM that has water as its main component. Although chitin-based materials can be readily constructed into networks that allow the free diffusion of water and bioactive molecules, these materials have not been comprehensively examined for their roles in the engineering of epithelial-derived organs and soft tissues. For decades, chitin-based materials have been known for their effects in promoting wound healing [33–36]. This application exemplifies a typical use of chitin-based materials in recovering epithelium and soft tissue. Recently, an increasing number of studies have demonstrated the potential of chitin-based materials in tissue engineering of epithelial and soft tissues. These studies extend the scope from simple epithelial reunion to the regeneration of many types of related tissues. This review will focus on the latest developments in the applications of chitin-based materials in tissue engineering by presenting some representative progress in the tissue regeneration of epithelial-derived organs and soft tissues.

## 2. Scaffolds in Tissue Engineering

### 2.1. Chitin-based Scaffolds

Tissue engineering combines the use of cells, bioactive factors, and supporting structures to regenerate and substitute damaged tissue structures and repair functionality. For tissue specificity, using appropriate materials for scaffold construction is imperative. An ideal material used for fabricating tissue-engineered scaffolds is expected to provide both structural support and control of bioactivity. In this regard, chitin-based materials are regarded as appropriate candidates because of their biodegradable properties and ability to be easily fabricated into distinct types of tissue scaffolds [11,37]. In addition, it is likely that chitosan can enhance cell function and regulate biomolecules [37,38]. Physical entrapment, chemical immobilization, or the grafting of bioactive factors in engineered chitin-based biomaterials can generate biologically active scaffolds.

Chitin-based materials can be readily fabricated into many forms of scaffolds, including hydrogels, microcapsules, membranous films, sponges, tubes, and a variety of three-dimensional porous structure [15,39]. Chitosan hydrogel can be prepared and crosslinked either covalently or ionically. Covalent crosslinking provides chitosan hydrogel with a permanent architecture that can be used to absorb and release bioactive factors or water. In contrast, ionically crosslinked chitosan hydrogel is formed by using reversible linkages. Because ionically crosslinked chitosan hydrogel can be dissolved, it is generally regarded as a tissue-tolerated scaffold. Moreover, chitin-based scaffolds can be generated in the form of an interconnected porous architecture [40]. Using the internal bubbling process, the size, distribution, and morphology of the pores within chitosan scaffolds can be easily controlled. The three-dimensional porous chitosan structure can be applied to a variety of tissue scaffolds. This structure resembles the tissue structure of several solid organs, and can be used to create regenerative scaffolds [41]. When chitin-based materials are fabricated into tubular forms, they can be successfully applied in the tissue engineering of nerves and blood vessels [42–44]. Chitin-based scaffolds are therefore versatile and can be optimized for various regenerative purposes [45].

In epithelial and soft tissue engineering, generating scaffolds with porous structures is important. Instead of mechanically strong properties, the reproduction of a flexible and plastic nature is required for regenerating epithelial and soft tissues. Chitosan can be fabricated into a porous structure that allows cells to be seeded. The space created by the porous structure facilitates cell proliferation, migration, and nutrient exchange. In addition, the controllable porosity of chitosan scaffolds is beneficial to angiogenesis, which is important in supporting the survival and function of regenerated soft tissue [11,46]. Furthermore, various types of glycoproteins and proteoglycans exist in the ECM of soft tissue, and most have anionic properties that can easily interact with chitosan [47]. These molecules are capable of binding to and regulating many bioactive factors; thus, this activity contributes strongly to the advantageousness of chitosan when used as a scaffold substrate in tissue engineering. Because chitosan resembles GAG in structure, it has been suggested that chitosan might mimic GAG in regulating and modulating many bioactive factors [48]. These characteristics are also helpful in retaining bioactivity within a tissue scaffold during *in vitro* cell seeding or *in vivo* implantation.

Based on these properties, chitosan scaffolds have been widely used in epithelial and soft tissue engineering [33,35,38,49,50]. The chitosan scaffolds show both cytocompatibility *in vitro* and biocompatibility *in vivo* [25]. Generally, chitosan evokes only a minimal foreign body reaction *in vivo*, and implanted chitosan scaffolds seldom induce chitosan-specific reactions [37]. Although many migratory neutrophils appear initially, few inflammatory reactions to chitosan occur. In addition, tissue-specific matrix deposition and active angiogenic activity are observed within implanted chitosan scaffolds [37]. These results provide evidence of the utility of chitosan in fabricating implantable scaffolds for tissue regeneration.

### 2.2. Blended Chitin-based Scaffolds

Despite positive characteristics, chitin-based materials have some disadvantages regarding tissue regenerative purposes. Cell seeding is a critical step in constructing bioengineered tissues. For some cells of specific tissue types, chitin-based biomaterials do not provide a friendly interface for cell adhesion [51–53]. Therefore, other biomaterials, such as collagen or fibronectin with tissue-specific binding sequence, should be blended with chitosan to produce scaffolds with higher cell affinity [52,53]. In addition to cell attachment, chitosan also restrains the migration and movement of certain types of cells. In tissue development, cell migration plays a major role in establishing tissue structure. Therefore, other materials that are more appropriate for directing the desired cell behaviors should be used [54,55]. The maintenance of cell survival and phenotypes, and the direction of cell differentiation are also critical to biomaterial selection. Chitosan has been shown to promote cell survival and neurite outgrowth when incorporated with poly-D-lysine [56]. Moreover, because chitosan usually lacks sufficient mechanical strength, it is either replaced by other substrates or blended with other polymers when used in tissue engineering of the skeletal system. Although mechanical strength is not of major concern in the engineering of epithelial and soft tissues, the requirement of appropriate tissue stiffness, as needed in connective tissue, still limits the utility of pure chitosan scaffolds. Because different tissues and cells vary in morphology, function, and structure, scaffold selection and fabrication should be well-selected and customized.

Many tissue-specific requirements benefit from blended chitosan scaffolds. Blends of different polymers can exhibit a wide range of chemical, physical, and biological properties in generating a substrate for tissue-specific regeneration. Chitosan has shown its utility as a polymer that can be blended with other materials to generate tissue-favorable scaffolds. These blended scaffolds show favorable mechanical, chemical, and biological properties while preserving the unique characteristics of chitosan. In ligament regeneration, chitosan-hyaluronan hybrid polymers can provide appropriate environments for cellular adhesion, proliferation, and ECM production, as well as facilitate the biological effects of seeded cells [57]. To mimic the morphological and mechanical properties of blood vessels and improve long-term patency rates, collagen has been crosslinked with chitosan to generate a tubular scaffold. This biocompatible scaffold demonstrated desirable porosity and pliability, and enhanced cell adhesion, proliferation, and ECM production [58,59]. In addition to vascular applications, chitosan/collagen blended scaffolds have also been employed in adipose tissue regeneration. When adipocytes were seeded, the *in vitro* cytocompatibility and *in vivo* biocompatibility of scaffolds were confirmed experimentally. Vascularization induction and adipose tissue formation were also observed [60]. When a blended chitosan scaffold was prepared with gelatin, the scaffold possessed the proper swelling property and burst strength, and it showed an appropriate environment for the growth and spread of vascular smooth muscle cells [44]. When mixed with silk fibroin, blended chitosan scaffolds exhibit increased ultimate tensile strength, elastic modulus, and water capacity [61]. This scaffold has been successfully applied in musculofascial tissue engineering to repair an abdominal wall defect [62]. Together, these representative blended chitin-based scaffolds demonstrate greater porosity, greater water uptake, and improved cellular adhesion for tissue-specific requirements (Table 1).

### 2.3. Chitosan Scaffolds Loaded with Bioactive Molecules

To increase the efficacy and completeness of tissue regeneration, several tissue engineering strategies have been employed, including changing the structure or the porosity of the scaffold [67,68], modifying the scaffold by introducing ECM elements [69,70], seeding the scaffold with cells [70,71], and applying bioactive molecules [72,73]. Among these strategies, the use of a scaffold containing bioactive molecules such as growth factors may be useful for facilitating tissue regeneration. Using this method, molecules that play an important role in guiding and modulating tissue regeneration can be delivered in a controlled manner to the exact site where cell differentiation and proliferation are expected to occur [74]. To this end, the *in vivo* efficacy of growth factors is critical, and the technology of drug delivery, particularly those designed in controlled-release formulations, is more suitable. Therefore, many controlled-release systems have been applied in fabricating tissue-engineered constructs to deliver essential growth factors and nutrients.

Structurally, chitosan is an unbranched, long-chained polymer that is composed of repeated disaccharide moieties. Therefore, it is generally considered to resemble the structure of GAG and believed to mimic its function in the modulation of cell behaviors and phenotypes [48]. Based on the cationic nature of the chitosan polymer, an ionic complex can be formed between chitosan and specific bioactive compounds. This interaction leads to the spontaneous formation of polyelectrolyte complexes by establishing strong, but reversible links. The temporary polyelectrolyte complex networks formed without covalent cross-linkers are more biocompatible, sensitive, and easily controlled. These features render chitosan a competent material for loading and releasing bioactive factors in a precisely controlled manner [1,23,75], and chitosan can be widely applied to the delivery of proteins, peptides, drugs, and extracellular components.

In addition to being positively charged, chitosan pocesses structurally intriguing properties that allow the introduction of desired biological function. Chitosan has abundantly reactive hydroxyl and amino units and can be functionally modified to increase biomaterial diversity. The biological activity beneficial to tissue regeneration can be introduced through the entrapment of bioactive agents in the scaffolds through physical adsorption [15]. Moreover, molecules of interest, such as peptides or proteins, can be incorporated into the chitosan scaffold by the formation of imide bonds [76,77]. The amine groups in the chitosan structure can be used to prepare different reactive chitosan derivatives. For example, trimethylated chitosan has been reported to be efficient in gene transfection without increasing cytotoxicity [78]. A chitosan tube immobilized with laminin peptides can facilitate proximal nerve sprouting and regenerate axon bridging [79]. These modifications render chitin-based materials more diverse and functional as well as facilitate the development of bioactive and tissue-friendly scaffolds for tissue regeneration.

Chitin-based materials have been described in the fabrication of tissue-engineered constructs conjugated with bioactive factors. For example, a chitin-based scaffold has been loaded with fibroblast growth factor 2 (FGF2) for periodontal tissue regeneration. The scaffold promoted structure formation, cell proliferation, and the mineralization of regenerated tissue [73]. Similar effects have been shown in accelerating wound healing [80]. A mixture of chitin-based hydrogel infused with a recombinant human epidermal growth factor (EGF) was effective in increasing local EGF concentration, enhancing keratinocyte differentiation, and facilitating the wound healing process [81]. Similar effects have been demonstrated in chitin-based scaffolds incorporating FGF2 [8] or platelet-derived growth factor (PDGF) [82]. Likewise, vascularization has been promoted using chitin-based scaffolds mixed with FGF2 [83]. Furthermore, the biological effects of DNA plasmids have been delivered using chitin-based scaffolds. When a vascular endothelial growth factor (VEGF) plasmid was incorporated into a chitin-based scaffold, a greater density of newly-formed and mature vessels was generated in a porcine model of skin defects [84]. In a nerve regeneration study, interferon-gamma (IFN-γ) was immobilized onto a chitin-based scaffold to induce differentiation of neural progenitor cells [72]. The same results were observed in periodontal tissue regeneration by incorporating transforming growth factor-beta 1 (TGF-β1) into a chitosan scaffold [85]. All cases show the versatility of chitin-based materials in cooperating with numerous factors to increase bioactive function during the regeneration of epithelial-derived organs and soft tissues (Table 2).

## 3. Biological Modulation in Tissue Engineering

### 3.1. Cell Preservation

Regulating cell phenotypes in a controlled manner is critical to the success of tissue regeneration-related biotechnology. When functional cells are isolated, the applied methodology is expected to preserve the inherent original phenotypes to maintain cell function. If pluripotent cells are used, their stemness and multi-functionality should be maintained during the processing procedure. However, when these cells are applied to the target organ, the desired differentiated status is expected to be induced and fulfill tissue functions. Therefore, the role of biomaterials in regulating and maintaining cell phenotype is the main issue of current research in tissue engineering.

Chitin-based materials have demonstrated their potential in maintaining and inducing cell phenotypes. When chitosan was used in culturing melanocytes, the unique phenotypic features of melanocytes were retained [86]. Similar results were also observed in culturing corneal keratinocytes [87] and skin keratinocytes [81]. For anterior cruciate ligament (ACL) cells, cell function could be induced with chitosan [88]. Likewise, differentiation of mucociliary epithelial cells originating from the respiratory tract was promoted by chitosan [53]. In addition, chitin-based materials were also capable of maintaining the stemness and inducing the differentiation of many progenitor cells. Chitosan scaffolds have been employed in neural stem cells (NSCs). When different forms of chitosan scaffolds were used, distinct differentiated phenotypes of NSCs could be controlled. NSCs cultured on films differentiated into astrocytes, whereas NSCs cultured on microconduits increased neuronal differentiation. In a chitosan porous structure, NSCs differentiated toward neurons [89]. When a chitin-based scaffold was used for cultivating adipose tissue-derived stem cells (ADSCs), stem cell characteristics and pluripotency were preserved [90]. Pluripotency was also preserved in embryonic stem-(ES) like cells *in vitro* [91]. When stem cells were co-cultured with endothelial cells on chitin-based scaffolds, these cells demonstrated characteristics affecting cell adhesion and migration [92]. Based on these studies, chitosan shows great promise in the development of tissue-specific scaffolds for regulating cell phenotype.

### 3.2. Regulation of Gene Expression

In tissue engineering approaches, biomaterials that not only support cell attachment and proliferation but also induce cell-inherent function are appealing. Most cell functions are controlled by gene expression, which mediates the production of essential factors for desired biochemical functions. Although gene modulation using the methods of molecular biology prevails, biosafety remains an issue when these methods are employed in clinical applications. The use of biomaterial provides another option. By introducing appropriate materials, cell response and gene expression can be regulated. For tissue regeneration, this technique may be more convenient and efficient.

The capacity of chitin-based materials for regulating gene expression has been documented in bone and cartilage tissue engineering [93]. In addition, growth factors were released from activated human platelets after chitosan stimulation [94]. Effects relevant to epithelial tissue engineering were initially demonstrated by the effect of chitosan on wound healing. After the application of chitosan, macrophages were stimulated to produce TGF-β1 and PDGF. In addition, specific ECM components, including fibronectin and type I and III collagens, were produced in cultured fibroblasts [95]. Similar functions were exhibited in soft tissue engineering by upregulating TGF-β1 in seeded anterior cruciate ligament cells [96].

### 3.3. Bioactive Molecule Regulation

Chitosan can react with several molecules and substrates via electrostatic and hydrophobic interaction. In this way, the functions of various bioactive molecules have been significantly modulated by chitosan. For tissue genesis, a repertoire of factors acts on cells in a temporally and spatially controlled manner. Therefore, providing tropic and tissue-specific molecules is critical for the success of tissue regeneration. Exogenous supplementation of bioactive factors usually has a short reaction time and loses bioactivity quickly; thus, it has been difficult to achieve the expected effects consistently. By using chitin-based materials as supporting scaffolds, these disadvantages can be surmounted and minimized. In addition to harnessing the endogenous activity of these factors, functional enhancement also can be mediated by chitosan in some applications.

**Figure 2.**
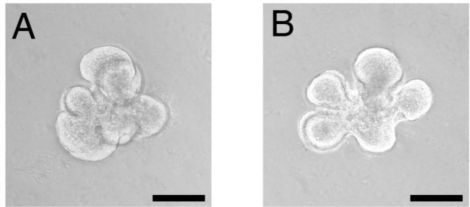
Salivary explants cultured with chitosan demonstrated remarkable lobular formation (**A**) and development of duct-like structures (**B**) (Scale bar: 100 μm). (Adapted from [12]).

In the salivary gland, the morphogenetic efficacy of mesenchyme-derived growth factors is dramatically augmented with the assistance of chitosan. The effects of epithelial morphogenetic factors, such as fibroblast growth factors 7 (FGF7), fibroblast growth factor 10 (FGF10), and hepatocyte growth factor (HGF), have been upregulated in the presence of chitosan. These factors promoted specific epithelial morphogenesis and proliferative and chemotactic activities [12] (Figure 2). The synergy between FGF7 and chitosan, which recruited mesothelial cells more extensively, has been applied to reduce pericardial adhesions [97]. When FGF-2 was prepared with chitosan, the retained FGF-2 remained biologically active with a prolonged half-life. Chitosan can protect FGF-2 from inactivation and thus prolonged FGF-2 activity [10]. EGF administered with chitosan can maintain keratinocyte differentiation with increased local concentration [81]. Therefore, by intensifying the bioactive factors in tissue genesis and promoting functional recovery, chitin-based materials can regulate key factors, expanding its role from a structural-supporting scaffold (Table 3).

### 3.4. Extracellular Matrix Production and Deposition

The production and deposition of tissue-specific ECM is required for cell survival, organization, and tissue genesis. Therefore, biomaterials used for tissue regeneration are expected to modulate ECM-producing cells. Moreover, tissue regeneration can be mediated through supporting materials that facilitates the production and deposition of ECM proteins in a tissue-specific manner [98]. Thus, materials capable of promoting ECM production are required for tissue regeneration.

**Figure 3.**
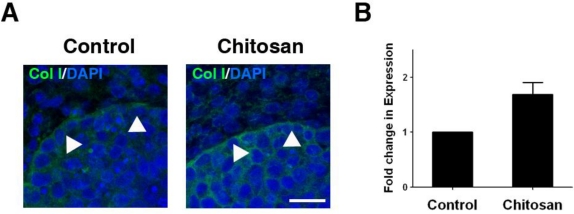
Extracellular matrix distribution and synthesis in salivary gland explants cultured with chitosan. (**A**) Distribution of type I collagen (green) in the epithelial-mesenchymal interface (arrowheads) of cultured salivary explants with and without chitosan (DAPI: blue; Col I: type I collagen; Scale bar: 20 μm); (**B**) The relative expression level of type I collagen was demonstrated using quantitative PCR.

Chitosan can stimulate cultivated cells to produce ECM (Table 4). In wound healing studies, chitin-based materials induced collagen fiber formation [99,100]. Type I, III and IV collagens were largely synthesized in implants made of chitin-based materials [101]. Although some studies have indicated that chitosan does not directly accelerate ECM production by cells, the increased synthesis of ECM-related growth factors might promote ECM production [95]. In the formation of intricate tissue structures, the presence of specific types of ECM is a requisite for organizing tissue architecture. The salivary gland is an epithelial-derived organ characterized by ramified structures; the concisely controlled appearance of specific components of the ECM is critical for morphogenesis [102]. With the assistance of a chitosan scaffold, the process could be accelerated *in vitro* [98]. Chitosan is a bioactive material used by the cell to synthesize and deposit essential ECM, which facilitates ensuing branch formation [98] (Figure 3). In joint connective tissue, cells cultured on a chitosan scaffold dramatically increased collagen production, further confirming that ECM production by cultured cells can be regulated by chitosan scaffolds [54,57,103]. Isolated human mesenchymal stem cells cultured on a chitosan scaffold showed an increased rate of collagen production [104], similar to the results of an *in vivo* study [37,62].

Other ECM components required for tissue regeneration are affected by chitin-based scaffolds. For instance, in cultured human venous fibroblasts, a chitin-based engineered construct demonstrated increased GAG content after culture [58]. GAG is an important component of soft tissue. These components can interact with protein to form proteoglycans, which can retain water molecules and increase water content. GAG also serves as a reservoir of essential bioactive factors [12]. Accordingly, by playing a role in regulating the synthesis of major ECM elements, chitin-based materials assist seeded cells in regenerating tissue. By creating a biomaterial closely resembling the native ECM environment, chitin-based materials show attractive features for epithelial and soft tissue engineering.

## 4. Applications

### 4.1. Chitin-based Materials in the Tissue Engineering of Surface-lining Layers

The body surface needs to be covered by specific tissue layers to establish a protective barrier against environmental threats. The outermost layer of the animal body consists of epithelial-derived tissue. The main function of an epithelial organ is to provide protection and facilitate secretion. Clinically, defects created by skin loss, such as large burns or chronic wounds, have remained challenging until recently.

Injured skin needs to be immediately covered with a dressing to restore tissue integrity, maintain homeostasis, and prevent the invasion of microorganisms. An optimal dressing should establish a barrier to environmental irritants, maintain a moist environment, and allow gaseous exchange. Therefore, biomaterials that possess the properties of biocompatibility, non-allergenicity, promotion of wound healing, and antimicrobial activities are regarded as ideal candidates. Chitin-based materials have been widely used as materials in the repair of skin tissue defect. Many features of chitin-based materials confer advantages over other materials for skin regeneration, including the promotion of cell infiltration, ECM production, antimicrobial properties, and maintenance of homeostasis [33,35,50,105]. In addition, chitin-based biomaterials demonstrate superior capacity in tissue adhesion and gas permeability, which helps to accelerate wound healing. Moreover, quaternary ammonium groups that are present within chitin-based materials efficiently interact with the cell. Thus, chitin-based materials possess high competence against microorganisms [106]. Chitosan can also be helpful during the different phases of wound repair [99]. The competence of chitosan in maintaining homeostasis is beneficial during the inflammatory stage, and its ability to stimulate fibroblast proliferation and inflammatory cell migration are also helpful for re-epithelization. Therefore, chitin-based materials and their derivatives have been made in a variety of forms for wound dressing [105]. Because of their antibacterial capability, chitin-based materials are fabricated into bandages [107]. These bandages are now regularly utilized from the bedsides to the battlefield, and are available both commercially and industrially [108]. Currently, similar products are being tested in clinical trials of wound debridement, chronic ulcers, and dental dressings. Therefore, because of their favorable biological and physicochemical characteristics, chitin-based materials are considered useful substrates for promoting the recovery of defective surface linings.

In the early developmental stages of skin tissue engineering, collagen-based scaffolds, which are composed of essential compounds of connective tissue, were initially used [109]. However, these scaffolds were obtained via chemical crosslinking for the acquisition of mechanical and material properties. With chitosan, a similar scaffold can be fabricated without using any chemical reagent [110]. In terms of functional characterization, the blended scaffold is comprehensively helpful for dermal reconstruction. Newly-formed ECM can be generated using macromolecules, such as collagen, fibronectin, and elastin, and an organized architecture can be established. Furthermore, a differentiated epidermis with multilayers is formed by expressing essential molecules in the dermo-epidermal junction [110]. This scaffold is regarded as a successful skin equivalent and shows great potential in cosmetic dermatology and for pharmaceutical uses [110].

Drug-carrying capacity of chitin-based scaffolds has also been applied to the fabrication of engineered skin scaffolds. These chitin-based scaffolds are fabricated and heparinized for angiogenic factor delivery. The heparinized scaffold has shown an enhancement of angiogenesis after *in vivo* implantation [59]. When FGF2 was added, the wound healing rate increased [80]. The results demonstrate that loading chitin-based scaffolds with angiogenic factors has potential use in developing a vascularized artificial dermis. Based on these experiences, the future development of engineered skin scaffolds will require the release of several essential molecules in a well-controlled manner, allowing engineered tissue equivalents to mimic native skin and increase their clinical utility [63,109,111].

To engineer an artificial product that can substitute for real skin, it is necessary to regenerate appendages and their associated skin features. The ideal engineered skin tissue should be pigmented to protect against irradiation. One skin disease amenable to treatment with tissue engineering is vitiligo, a common disfiguring and depigmenting disorder that destroys melanocytes. Extensive vitiligo lesions require melanocyte transplantation. Improved melanocyte transplantation is achieved by culturing melanocytes on appropriate biomaterials to form a cellular patch. Chitosan-containing scaffolds support the growth and phenotypic maintenance of melanocytes. It has been demonstrated that melanocyte spheroids, which retain melanocyte viability, were formed on a chitosan-coated surface. When cells were reinoculated onto other surfaces, the cells regained their dendritic phenotypes. These results show that a three-dimensional culture system constructed of chitosan may serve as a cellular patch for vitiligo treatment [86].

In addition to skin regeneration, organs located on the body surface, such as the eyes, also require regenerating coverage when a defect occurs. Corneal defects are major clinical issues in ocular surface reconstruction. For cornea regeneration, scaffolding methods can promote corneal epithelial healing and preserve native cell phenotypes. Clinically, an amniotic membrane is routinely used as a scaffold to promote corneal epithelial wound healing and preserve the original phenotypes of corneal epithelial cells [112]. When chitosan is prepared in a membranous form to culture corneal epithelial cells, it shows a similar capacity in supporting the growth of cultured corneal epithelial cells. With chitosan, cells can grow without any change of their endogenous characteristics. Chitosan also assists in preserving the phenotypes of the keratinocytes, which are the primary cells on the ocular surface that regulate stromal ECM and improve wound healing. Cultured on a chitosan surface, corneal keratinocytes formed in spheroid shapes that were beneficial in preserving the original features for subsequent transplantation [87]. The spheroids were generated by cell aggregation instead of cell proliferation and had a higher capacity for regeneration. These results suggest that the use of chitin-based biomaterials is another choice for treating ocular surface disorder [113].

Similarly, in the aerodigestive system, chitin-related biomaterials are useful for tissue engineering. By coculturing human oral epithelial cells and fibroblasts in a porous collagen–glycosaminoglycan–chitosan scaffold, an oral mucosa equivalent was engineered [65]. The engineered mucosa tissue resembles human native oral mucosa manifested by nonkeratinized full-thickness tissue structure. The tissue-engineered human oral mucosa can be used to close mucosal wound resulting from disease or medical therapy. Most surfaces in the respiratory tract are covered by ciliated respiratory epithelium. Without this epithelial lining, occurrences of inflammation, bleeding, and nasal crusting might ensue [114]. A tissue-engineered scaffold could provide physical support and regulate respiratory epithelial cell growth, adhesion, and differentiation. Collagen was initially used in constructing aerodigestive epithelia because of tissue similarity [115]. The unsuitable degradation rate and mechanical strength of collagen have made its application infeasible [116]. When collagen is blended with chitosan to prepare a scaffold, it improves biocompatibility and promotes mucociliary differentiation in nasoepithelial cells [53]. Chitosan also increases the mechanical strength of collagen-based scaffolds and facilitates cultured cells in expressing tissue-specific epithelial markers [117]. Accordingly, chitin-based materials are appropriate grafting materials for airway epithelia and can be used as scaffolds in engineering airway linings for respiratory system regeneration.

### 4.2. Chitin-based Materials in Tissue Structure Formation

In addition to outer surface linings, many tissues have unique cellular architectures that are organized in well-designed tissue patterns. With these unique structures, tissue-specific physiological functions can be efficiently fulfilled. For instance, many tissues are characterized by a branching structure, which helps to create the larger cellular surfaces that are necessary for metabolic requirements. In embryonic development, ramified structures are generally generated by branching morphogenesis [118,119]. The salivary gland, an organ responsible for saliva secretion and regulation, is typically formed by this process. To engineer this unique tissue structure, an appropriate scaffold that helps to recapitulate the branching processes must be found. For this purpose, chitin-based scaffolds can provide preferential characteristics for enhancing salivary gland branching [98]. By interacting with cultured salivary tissue, chitosan can induce salivary cells to synthesize and deposit collagen-related ECM, which is beneficial to branch formation. Other biocompatible and biodegradable biomaterials, such as lactic-co-glycolic acid and poly epsilon-caprolactone, do not support branch formation and decrease cell migration and viability [120]. Thus, chitin-based biomaterials are superior substrates for enhancing the branching morphogenesis of the salivary gland.

In addition to facilitating ECM production for branching morphogenesis, chitosan promotes epithelial-mesenchymal interactions during organogenesis. During tissue formation, a reciprocal interaction between the epithelium and mesenchyme is required [121]. Using chitosan, the efficacy of mesenchymal morphogenetic instruction toward salivary epithelium is promoted, generating that particular tissue structure more efficiently [12]. In the repertoire of tissue formation, the tissue-specific morphogenetic effect of mesenchyme-derived factors, such as FGF7, FGF10, and HGF, are all reinforced. In addition, the proliferative and chemotactic properties of these morphogens are enhanced. These results demonstrate the promising potential of chitin-based biomaterials in regenerating tissue structure. By comprehensively intensifying the essential mesenchyme-derived growth factors, chitosan can facilitate the formation of salivary epithelial architecture. These results demonstrate the novel functions of chitosan, serving as a scaffold for tissue structural formation and as a regulator of morphogenetic factors. Both functions are essential in organogenesis [12].

Chitosan’s ability to promote tissue structure formation is specific, unique, and largely affected by molecular weight and specific chemical linkages [122]. Furthermore, chitosan can independently induce salivary branching under serum-free conditions [12]. In previous reports, serum was required for tissue interactions between the salivary epithelium and mesenchyme [123,124]. The supplementation of serum is beneficial to salivary morphogenesis because it provides many essential factors [125]. However, for tissue regeneration, the addition of serum might be costly and impair biocompatibility. These results suggest a novel role of chitosan in reducing the need for serum in tissue engineering, an important step towards future clinical use.

### 4.3. Chitin-based Material in Connective Tissue Engineering

Within the body, connective tissue plays a role in maintaining contour and shape, surrounding or separating organs, relaying contractile forces, and modulating physiological functions. There is evidence that chitin-based materials are implicated in the regenerative applications of connective tissue. In the joints of the extremities, ligaments are important tissues to maintain body stability and movement. From a tissue engineering perspective, the use of a scaffold is necessary for ligament regeneration to help related cells reestablish a similar tissue environment [88,96,126]. When human ACL fibroblasts were cultured with chitosan, a specific gene profile that regulates matrix production was up-regulated. This advantage compensated for insufficient matrix production by native well-differentiated ACL cells. Without supplemental chemical reagents, chitosan-based biomaterials can specifically control cellular morphologies, regulate gene expression, and increase protein production [96].

Similar effects are observed when chitin-based scaffolds are used to promote periodontal tissue regeneration. In blended chitosan scaffolds loaded with FGF2, both periodontal ligament cells and cementoblasts have demonstrated vigorous proliferation and migration [73]. A blended chitin-based scaffold has been used to repair a musculofascial defect. The abdominal wall defect was remodeled, showing seamless integration with adjacent native tissue and mechanical strength similar to native tissue [62]. Together, these results indicate that chitin-based materials could provide a cell-favorable environment for connective tissue regeneration.

### 4.4. Chitin-based Material in Nerve Tissue Engineering

To repair peripheral nerve injuries with neural gaps, the current standard treatment uses an autologous nerve graft to bridge the neural gap and facilitate nerve regeneration and reconnection. Engineered nerve grafts are usually composed of a neural scaffold, seeded supportive cells, and growth factors [127]. Among the various biomaterials under investigation, scaffolds made of chitin-based materials have drawn much attention [128]. Chitin-based materials support neuronal growth [129]. In addition, many different substrates and bioactive molecules have been added into chitin-based scaffold to increase their affinity with nerve cells [130–133]. For examples, polyglycolic acid fibers added to a chitosan nerve scaffold were used to reconnect a long sciatic nerve gap in a dog model and a long median nerve gap in a clinical study [134,135]. Furthermore, when the scaffold was seeded with bone marrow mesenchymal cells, a longer neural gap of up to 50 mm in length was repaired in a dog sciatic nerve [136]. Therefore, a chitin-based, nerve-guiding scaffold can successfully connect long gaps and promote nerve regeneration and functional recovery.

In nerve regeneration, Schwann cell (SC) supports neurite outgrowth by releasing neurotrophic factors, expressing neuron-specific ligands, guiding neurite outgrowth, and producing and depositing ECM [137]. A chitosan scaffold can support SC adhesion, migration, and proliferation [138], and induce SC alignment, leading to oriented axonal growth and preventing neuroma formation [139]. In addition, chitosan scaffolds have sufficient mechanical strength to maintain the conduit space, provide a favorable environment for cell migration and attachment, and improve the permeation of neurite-related factors [43]. Because of the SC alignment along the scaffolds, oriented fibrous sheets were established. After transplantation *in vivo*, vigorous sprouting myelinated axons were found and nerve function was recovered. These results demonstrate that engineered nerve conduits made of chitin-based materials comprehensively support neurite regeneration.

### 4.5. Chitin-based Material in Adipose Tissue Engineering

Adipose tissue defects resulting from congenital abnormalities, trauma, senile alteration, or medical treatments significantly affect patients’ health, appearance, and quality of life. The current therapeutic modalities that include primary closure, flap transplantation, or material implantation are not ideal given clinical requirements. The main goal of adipose tissue engineering is to design a scaffold that mimics native adipose tissue and maintains a three-dimensional volume following implantation [140,141]. For this purpose, chitin-based materials have been used for adipose tissue engineering. Prepared in a blended form, the scaffold could provide appropriate micro-architecture and water holding capacity. When seeded with cells, biocompatibility has been confirmed both *in vitro* and *in vivo*. After transplantation into animals, the scaffolds induced vascularization and successfully generated adipose tissue [142]. Accordingly, these reports demonstrate the feasibility of applying chitin-based scaffolds to adipose tissue engineering.

### 4.6. Chitin-based Material in Vascular Tissue Engineering

In many engineered tissue equivalents, an established vascular system is critical to the ensuing survival and functionality of the engineered organs. For survival of transplanted tissue, angiogenesis and neovascularization must occur. In addition, transplantation with vessel grafting is needed to treat vascular diseases. Therefore, materials that serve as vascular grafting substitutes or induce angiogenesis have been of interest for decades [143].

Chitin-based scaffolds have been widely investigated for use in constructing vascular substitutes. Chitosan promotes endothelial cell migration in wound healing [144]. Blended collagen and chitosan scaffolds hold further promise as scaffolds for vascular tissue engineering [58,145]. *In vitro* studies confirm the capacity of these scaffolds in supporting cell attachment, growth, and proliferation. These scaffolds have mechanical properties similar to native blood vessels and superior biocompatibility after transplantation. Moreover, chitin-based materials can facilitate ingrowth of connective tissues which promotes neo-vascularization after implantation [145]. When GAG is incorporated into chitosan, its effects on inhibiting vascular smooth muscle cell growth and coagulation counteract the difficulties of incomplete endothelialization and muscle hyperplasia [146]. Moreover, heparin–chitosan scaffolds can be applied in vascular tissue engineering because heparin is effective in reducing thrombosis and recruiting related growth factors [83,147,148]. *In vivo* results have demonstrated that the heparin–chitosan scaffolds can stimulate cell proliferation to form a thick vascularized granulation [146]. These studies demonstrate the feasibility of applying composite chitin-based scaffolds in vascular tissue engineering.

In addition to acting as scaffold components, chitin-based materials can deliver bioactive molecules for vessel regeneration [83,149]. When chitosan is prepared as a hydrogel, the incorporated angiogenic factors, such as FGF2, can be gradually released *in vivo* with hydrogel degradation. Retained in chitosan hydrogel, the activity of biomolecules remains stable. When tested *in vivo*, hydrogel injection results in a significant increase in newly-formed blood vessels and fibrous tissue formation. The controlled release of angiogenic factors from chitosan hydrogel effectively reconstructed collateral circulation and recovered ischemic limbs in animal models [150]. In addition, a gene-activated engineered tissue was developed using chitin-based materials. When VEGF DNA was incorporated into a blended chitosan scaffold, many newly-formed and mature vessels formed [84]. Thus, it has been proposed that chitin-based materials are potential delivery vehicles for bioactive factors in vascular tissue engineering.

## 5. Current State, Challenge, and Future Prospects

This review summarizes the utility of chitin-based materials in engineering tissues that originate from epithelium and soft tissue. Successful applications in several biomedical fields demonstrate that chitin-based materials have made a significant contribution to relevant tissue regeneration. Because of the favorable biological properties, including the preservation of cellular phenotypes, enhancement of bioactive molecules efficacy, gene regulation, and tissue-specific ECM production and deposition, chitin and chitosan-related materials are attractive scaffolds in regenerating epithelial-derived organs and soft tissues.

Recent research shows that chitin-based material can be used as a bioactive tissue engineering scaffold and acts as an efficient carrier of bioactive molecules. The results demonstrate the tremendous potential of chitin-based material for clinical applications. Nonetheless, until recently, most applications had been investigated only in the laboratory. Only a few products of chitin-based materials, such as dressings designed for wound care, have been successfully transferred from bench to bedside. The currently ongoing clinical trials of chitin and chitosan-related material products mostly focus on topical hemostatic control and metabolic regulation. For these clinical applications, chitin-based materials are fabricated into sponges or microcapsules to increase the surface for tissue interactions and enhance the efficiency of bioactive factor delivery. Chitin-based materials produced in spongy forms lack mechanical strength and precise control of their bioactive factor release. Materials designed for encapsulation only provide the capacity to load and release biomolecules and do not provide structural support for tissue regeneration [15]. It is likely that a variety of properties unique to chitin-based materials have seldom been applied in fabricating clinically applicable scaffold. Therefore, current progress in clinical studies is not as advanced as academic research.

In the engineering of epithelial and soft tissues, the accurate control of bioactive molecules, particularly those related to tissue development and morphogenesis, is imperative. During organogenesis, these key factors always appear in a precise spatial and temporal manner to direct tissue formation. Because of stringent regulation of the natural developmental blueprint, the ideal scaffold should be designed to release bioactive factors sufficiently and effectively. In addition, a variety of growth factors and morphogens are involved in tissue formation. Therefore, next generation scaffolds should be able to carry many different bioactive factors, and release them in specific order. To this end, decisions on how to control the separate loading capacity, kinetics of drug release, and rate of substrate degradation are the major challenges to be faced. Because of the unique features of chitin-based material that have been mentioned above, a chitin-related substrate might be the substrate with the greatest potential. Furthermore, because epithelial and soft tissues have an important stereo-architecture, the ability to transfer current knowledge regarding two-dimensional cell-biomaterial interactions into three-dimensions is required in developing a bulk scaffold for *in vivo* animal and clinical studies. In achieving these aims, the biologically attractive features of chitin-based materials can be fully utilized, and these may facilitate the transition of the technology from bench to bedside.

## Figures and Tables

**Table 1.** Different chitin-based scaffolds blended with natural polymers used in tissue engineering of epithelial organ and soft tissue.

**Scaffold**	**Application**	**Effect**	**Ref.**
Chitosan-hyaluronan	Ligament	Cellular adhesion	[57]
		Cell proliferation	
		ECM and production	
Chitosan/Collagen	Blood vessel	Cell adhesion	[58,59]
		Cell proliferation	
		ECM production	
Chitosan/Collagen	Adipose tissue	Vascularization induction	[60]
		Adipose tissue formation	
Chitosan/Collagen	Skin	Cell proliferation	[63]
		Cell infiltration	
Collagen-chitosan/fibrin glue	Skin	Cell growth	[64]
Collagen-glycosaminoglycan-chitosan	Oral mucosa	Cell differentiation	[65]
		Cell proliferation	
		ECM production	
Chitosan/gelatin	Skin	Cell survival	[66]
Chitosan/gelatin	Blood vessel	Cell growth	[44]
		Cell migration	
Chitosan/silk fibroin	Musculofascia	ECM deposition	[62]
		Vascularization	
		Cellular infiltration	

**Table 2.** Chitin-based scaffold loaded with bioactive molecules in tissue engineering of epithelial and soft tissue.

**Scaffold**	**Factor**	**Application**	**Effect**	**Ref.**
Chitosan-hydroxyapatite	FGF2	Periodontal tissue	Cellular structure formation	[73]
			Cell proliferation	
			Mineralization	
Chitosan hydrogel	FGF2	Wound healing	Wound healing, Angiogenesis	[8]
Chitosan hydrogel	FGF2	Vascularization	Angiogenesis	[83]
Chitosan	FGF2	Wound healing	Fibroblast proliferation, Vasculogenesis	[80]
Chitosan-Pluronic	EGF	Wound healing	Keratinocyte differentiation	[81]
			Wound healing	
Chitosan-collagen gel	PDGF	Wound healing	Wound healing	[82]
			Biomimetic effects	
			Chemotactic effects	
collagen-chitosan/silicone	VEFG	Vascularization	Angiogenesis	[84]
			Vasculogenesis	
Methacrylamide chitosan scaffold	IFN-γ	Nerve regeneration	Differentiation of neural progenitor cells	[72]
Chitosan/Collagen	TGF-β1	Periodontal Tissue	Tissue regeneration	[85]

**Table 3.** Activity modulation of bioactive molecules by chitin-based scaffolds in tissue engineering applications.

**Molecule**	**Tissue/Organ**	**Effect**	**Ref.**
FGF7	Salivary gland	Lobular formation	[12]
		Cell proliferation	
FGF7	Mesothelium	Synergistic effect	[97]
		Adhesion decrease	
FGF10	Salivary gland	Ductal elongation	[12]
		Cell proliferation	
HGF	Salivary gland	Cell migration	[12]
		Cell proliferation	
		Chemotaxis	
FGF2	Vasculature	Cell proliferation	[10]
FGF2	Periodontal tissue	Cell proliferation	[73]
EGF	Skin	Cell differentiation	[81]

**Table 4.** Production and deposition of ECM components by chitin-based scaffolds in tissue engineering of epithelial and soft tissue.

**ECM component**	**Tissue/Organ**	**Effect**	**Ref.**
Collagen, Type I	Skin	Wound healing	[101]
	Salivary gland	Branching morphogenesis	[98]
	Ligament	Cell proliferation	[57]
	Vessel	Cell phenotype	[58]
	tendon	Cell ECM production	[103]
Collagen, Type III	Skin	Wound healing	[101]
	Salivary gland	Branching morphogenesis	[98]
	Ligament	Cell phenotypes	[54]
Collagen, Type IV	Skin	Wound healing	[101]
Glycosaminoglycan	Vessel	Cell phenotype	[58]
